# Test Anxiety in Adolescent Students: Different Responses According to the Components of Anxiety as a Function of Sociodemographic and Academic Variables

**DOI:** 10.3389/fpsyg.2020.612270

**Published:** 2020-12-15

**Authors:** Rosa Torrano, Juan M. Ortigosa, Antonio Riquelme, Francisco J. Méndez, José A. López-Pina

**Affiliations:** University of Murcia, Murcia, Spain

**Keywords:** adolescents, text anxiety, age, gender, type of exam

## Abstract

**Objective:**

Test anxiety (TA) is a construct that has scarcely been studied based on Lang’s three-dimensional model of anxiety. The objective of this article is to investigate the repercussion of sociodemographic and academic variables on different responses for each component of anxiety and for the type of test in adolescent students.

**Method:**

A total of 1181 students from 12 to 18 years old (*M* = 14.7 and SD = 1.8) participated, of whom 569 were boys (48.2%) and 612 girls (51.8%). A sociodemographic questionnaire and the *Cuestionario de Ansiedad ante los examenes-Adaptado (CAEX-A)* [Test Anxiety Questionnaire-Adapted] an adaptation for Spanish secondary school levels (ESO) and Bachillerato were administered.

**Results:**

Girls scored higher on the cognitive and physiological components of TA than boys, the intensity of the physiological response increasing with age. Bachillerato level students reported more physiological anxiety than those of ESO level. Students with better marks in the previous year presented more anxiety in the cognitive component, while those who obtained the lower mark presented higher anxiety values in the behavioral component. Participants reported that the types of tests that cause them more anxiety were oral tests in front of the class, oral presentation in front of a panel, and mathematics tests.

**Conclusion:**

Adolescents show a differential response of TA based on the physiological, cognitive and motor components, mediated by the variables of gender, age, grade, academic performance and type of exam. These results serve to design specific intervention programs to manage anxiety in situations of academic assessment.

## Introduction

Test anxiety (TA) is the relatively stable tendency to generate a disproportionate emotional response in academic assessment situations, due to concern about poor performance and possible negative consequences (Balogun et al., 2017; Putwain and Symes, 2018). However, there is no consensus in the scientific and academic community on its conceptualization (Escolar-Llamazares and Serrano-Pintado, 2014). The lack of agreement is explained by the nature and dynamic structure of the construct and it is underscored by the diversity of terms used in its definition: emotion, worry, tension, lack of security, irrelevant thinking, cognitive reactions to assessment situations, off-task behavior, autonomic reactions, bodily reactions, thinking, self-rumination, affective response, cognitive response, psychophysiological response, and motivational response (Liebert and Morris, 1967; Pekrun et al., 2004; Sarason, 1984; Wren and Benson, 2004; Pena and Losada, 2017).

Since the beginning, different models have been proposed for TA: one-dimensional (Mandler and Saranson, 1952), two-dimensional (Liebert and Morris, 1967), and multi-dimensional (Hodapp and Benson, 1997; Wren and Benson, 2004; Chin et al., 2017), giving rise to various evaluation instruments (Osterhouse, 1970; Valero, 1999; Lowe and Lee, 2004; García-Fernández et al., 2011; Sung and Chao, 2014). Within the multi-dimensional models, the three-dimensional theory of anxiety proposed by Lang (1968) stands out, which establishes that anxiety is composed of three response systems: cognitive, physiological, and behavioral or motor, which can be manifested in a discordant and asynchronous way (Furlan, 2006; Martínez-Monteagudo et al., 2012).

Research on TA based on Lang’s three-dimensional model has focused on the university population (Valero, 1999; Rodríguez et al., 2014). However, there are some studies on non-university education levels, where differences have been found in socio-demographic variables such as ethnicity, age/grade level, and—especially—gender. The most consistent findings are that (1) girls have more TA at all levels of education (Putwain, 2007; Núñez-Peña et al., 2016; Aydin, 2017; Sari et al., 2017), which has been confirmed in different countries (Jalilian et al., 2016; Olaseni and Olomosaye, 2018; Brandmo et al., 2019; Lowe, 2019) and (2) in girls, the physiological and cognitive component of TA predominates, whereas in boys, it is the behavioral or motor component that prevails (Wren and Benson, 2004; Rodríguez et al., 2014; Aydin, 2019). Boys tend to see test situations as a challenge, and their reaction depends on their perception of their own competence in dealing with the task; if they consider themselves capable of successfully passing the test, they become involved behaviorally and emotionally, and if not, they give up, although in both cases they present low levels of TA (Rosàrio et al., 2008). Girls, on the other hand, interpret test situations as more of a threat and present higher levels of TA, which is expressed as fear, difficulty concentrating on the task, and poor academic self-concept/self-esteem (Spielberger, 1980). The gender difference in coping with test situations is probably due to the different degree of societal demand, which emphasizes the necessity for students to be involved in tasks as well as high expectations of success (Rosàrio and Soatres, 2003). This difference does not affect academic performance however, possibly because of girls’ use of appropriate coping strategies (Núñez-Peña et al., 2016).

The relationship between the type of test and TA has been approached mainly from the perspective of the student’s preference for the evaluation format. Zeidner (1987) found that most students prefer multiple-choice tests to essay tests, because the study and expression of ideas is more demanding in the latter type, which generates more TA. Similarly, Tas and Minaz (2019) found that 70.67% of students choose multiple choice tests for reasons of convenience, ease, and accuracy. In the same vein, van de Watering et al. (2008) concluded that students choose written assessments, especially in multiple choice format, because oral tests are more stressful and difficult to prepare. Finally, Núñez-Peña et al. (2016) studied the differences in TA according to the type of test and gender, finding that girls had more anxiety and that anxiety levels increased in both genders according to the following sequence: test with calculations, open-ended test, oral presentation. However, in none of the reviewed studies do the results take into account the individual components of TA.

Research in this field has proven that there is discordance and asynchronism among TA response systems. For this reason, this study proposes as a general objective to analyze TA based on Lang’s theory of the triple response system, considering the modulating role of sociodemographic and academic variables in adolescents. Specifically, the follow research questions were formulated:

1) Are there differences in the components of TA according to the gender and age of adolescent students?
2) Are there differences in the components of TA according to the educational level and academic performance of adolescent students?
3) Are there differences in the components of TA according to the type of test and the subject matter?
4) Are there differences in TA in the type of exam according to gender, age, and educational level?

## Materials and Methods

### Participants

The initial sample consisted of 1,409 students, of which 189 (13.41%) did not attend class on the days of data collection and 39 (2.76%) did not obtain parental consent. Finally, a total of 1,181 students participated, from two public secondary schools (IES) in the Region of Murcia (Spain), selected for convenience. The age range was 12–18 years (*M* = 14.7 and SD = 1.8). The age groups were formed according to the classification of Oliva (2004): early adolescence (12–14 years), middle adolescence (15–16 years), and late adolescence (17–18 years). In Spain, studies during adolescence are organized into two educational levels: (a) Obligatory Secondary Education (ESO in Spanish), four school years from 12 to 15 years of age and (b) Non-Compulsory Secondary Education (Bachillerato/BAC in Spanish) two school years from 16 to 17 years of age (Organic Law 8/2013, of December 9, for the improvement of educational quality; LOMCE) (see Table 1).

**TABLE 1:** Distribution of the sample according to sociodemographic and academic variables.

	***N***	**Percentage**
**Gender**		
Male	569	48.2
Female	612	51.8
Total	1,181	100
**Age group**		
Early adolescence (12–14 years old)	561	41.2
Middle adolescence (15–16 years old)	379	32.1
Late adolescence (17–18 years old)	241	20.4
Total	1,181	100
School year*		
1st ESO (7th grade)	246	20.8
2nd ESO (8th grade)	186	15.7
3rd ESO (9th grade)	228	19.3
4th ESO (10th grade)	203	17.2
1st BAC (11th grade)	186	15.7
2nd BAC (12th grade)	132	11.2
Total	1,181	100
Mark (previous year)*		
Failure (D)	184	15.6
Adequate (C-)	141	11.9
Good (C +)	311	26.3
Very good (B)	405	34.3
Excellent (A)	140	11.9
Total	1,181	100

*The equivalents in the United States academic year and grading system are indicated in parentheses.

### Instruments

The *Cuestionario de Ansiedad ante los Exámenes para ESO y Bachillerato – Adaptado* [Test Anxiety Questionnaire for ESO and Bachillerato – Adapted] (CAEX-A) was used, which is an adaptation for ESO and Bachillerato students of the *Cuestionario para la Ansiedad ante los Exámenes* [Test Anxiety Questionnaire] by Valero (1999). The first part consists of 37 items that evaluate the intensity of the TA responses with a 5-point Likert type scale (0 = not at all, 1 = a little, 2 = a moderate amount, 3 = a lot, and 4 = very much) and the second of 11 items on TA caused by different test modalities evaluated with the same scale, to which the option “I have not done it” was added in case the student had never taken that type of test.

The first part of the TA questionnaire is based on Lang’s three-dimensional theory of anxiety and comprises three factors: (1) physiological responses (20 items, range 0–80) (e.g., *in the test, my hands sweat. When I’m taking the test my heart beats very fast*); (2) cognitive responses (14 items, range 0–56) (e.g., I *think I’m going to get nervous and forget everything*); and 3) motor responses (3 items, range 0–12) (e.g., *I get sick and make excuses for not taking the test*). The total score is obtained by adding up the scores of the items of the three factors (range 0–148).

The internal consistency, Cronbach’s α, is high except in the behavioral dimension: CAEX-A (α = 0.94), physiological responses (α = 0.90), cognitive responses (α = 0.90), behavioral responses (α = 0.50). In addition, the value of omega exceeds 0.85 in all scores. Also the test-retest reliability shows high values in the correlations, which points to the temporal stability of the instrument: *r*_xy_ = 0.87 for the physiological response; *r*_xy_ = 0.81 for the cognitive response, *r*_xy_ = 0.52 for the motor avoidance response, and *r*_xy_ = 0.66 for the total score. The percentage of variance explained by each factor is as follows: 41.33 for the physiological, 4.85 for the cognitive, and 6.84 for the behavioral. Finally, the convergent validity of the instrument with the *State-Trait Anxiety Inventory for Children* (STAI-C; Spielberger, 1973; Spielberger et al., 1990) is *r*_xy_ = 0.62 for the total scale (Torrano et al., 2020).

The *Questionnaire of sociodemographic and academic variables.* An *ad hoc* instrument was developed to collect the sociodemographic and academic data of interest to the study, including gender, age, and average mark from the previous year.

### Procedure

Once permission was obtained from the Research Commission of the University of Murcia, the agreement of the schools was processed. Then, the authorization and informed consent was requested of the parents or guardians and the students themselves for participation in the study.

In agreement with the teachers, the moment chosen for its suitability to administer the questionnaires was a week of exams during the term’s evaluation period, two weeks before the end of term.

Four psychology graduates were trained to administer the questionnaires collectively in the classroom. The students first answered the socio-demographic questionnaire and then the assessment administrators gave the standardized instructions for the CAEX-A. The administrators read the item aloud and the students responded. During the administration they answered any doubts and supervised the completion to guarantee the independent and adequate answering of the questionnaire.

### Data Analysis

Since some of the assumptions of the parametric statistics were not confirmed, the Man–Whitney *U* test was used for two-group comparisons with one quantitative variable and the Kruskal–Wallis *H* test was used for three or more comparisons. In the latter case, the analysis was completed with pairwise comparisons using the Mann–Whitney *U*-test with Bonferroni-corrected *p*-value.

A Pearson correlation coefficient was established between TA in each type of exam and the global and partial scores of the CAEX-A. In addition, the same type of analysis was applied to determine the relationship between the age of the participants and anxiety components.

Finally, the differences in anxiety for each type of exam were studied according to gender, grouped age and educational level.

The *Statistical Package for the Social Sciences* (SPSS) version 22 (IBM Corp, 2011) was used for the statistical analyses.

## Results

### Test Anxiety, Gender, and Age

Girls’ scores were significantly higher, except on the behavioral component where boys scored slightly higher, but this was not statistically significant (see Table 2).

**TABLE 2:** Gender difference in test anxiety scores.

**Component**	**Sex**	**Average range**	**U**	**Z**	**Sig.**
Physiological	Boys	498.99	118,140.50	−9.250	<0.001
	Girls	682.03			
Cognitive	Boys	501.88	120,005.00	−9.092	<0.001
	Girls	682.09			
Behavioral	Boys	596.48	170,175.50	−0.752	ns
	Girls	584.08			
Total TA	Boys	494.54	115,991.00	−9.433	<0.001
	Girls	680.84			

Test anxiety scores increased with age, except in the cognitive component where the peak was reached in middle adolescence, but again this was not statistically significant (see Table 3).

**TABLE 3:** Age differences in test anxiety scores.

**Component**	**Age**	**Average range**	**H**	**df**	**Sig.**
Physiological	Early adolescence	545.55	17.832	2	<0.001
	Middle adolescence	608.92			
	Late adolescence	648.41			
Cognitive	Early adolescence	580.11	0.831	2	ns
	Middle adolescence	600.52			
	Late adolescence	591.56			
Behavioral	Early Adolescence	564.32	9.226	2	<0.05
	Middle adolescence	611.52			
	Late adolescence	618.51			
Total TA	Early Adolescence	557.57	7.054	2	<0.05
	Middle adolescence	602.48			
	Late adolescence	618.71			

Subsequent pairwise comparisons using the Mann–Whitney *U* showed that differences in the physiological component occurred between early and middle adolescence (*U* = 93,293.00; *p* < 0.01), and early and late adolescence (*U* = 55,188.00; *p* < 0.001). In the behavioral component, similar results were found to the previous ones, since differences appeared between early and middle adolescence (*U* = 97,756.00; *p* < 0.05), and early and late adolescence (*U* = 61,185.00; *p* < 0.05). Finally, the same pattern of results was repeated in the total score between early and middle adolescence (*U* = 95,866.00; *p* < 0.05), and early and late adolescence (*U* = 59,294.00; *p* < 0.05).

In addition to previous results, a Pearson correlation coefficient (Bootstrap with a 95% confidence interval) was performed between age and TA components, reaching a positive and significant relationship in the physiological *t* (*r*_*xy*_ = 0.128, *p* < 0.001) and behavioral component (*r*_*xy*_ = 0.09, *p* < 0.01) and the total scale (*r*_*xy*_ = 0.088, *p* < 0.01).

### Test Anxiety, Educational Level, and Academic Performance

Firstly, when comparing TA according to educational levels, differences were found in the total score and in the physiological component, where Bachillerato students scored higher in both cases (see Table 4).

**TABLE 4:** Difference between educational levels in test anxiety.

**Component**	**Educational level**	**Average range**	**U**	**Z**	***p***
Physiological	ESO/BAC	552.65/679.35	106,577.50	−5.70	<0.001
Cognitive	ESO/BAC	582.62/606.30	130,824.50	−1.06	ns
Behavioral	ESO/BAC	593.42/582.57	134,537.50	−0.58	ns
Total TA	ESO/BAC	564.84/637.28	118,152.50	−3.26	<0.01

Closely related to age, the results obtained with the grade were similar, that is, significant differences were found in the TA total score (*H* = 23.40, *p* < 0.001), in the physiological (*H* = 53.46, *p* < 0.001) and behavioral component (*H* = 20.56, *p* < 0.001), but not in the cognitive one (*H* = 7.25, n.s). Thereby, the scores on total TA and the physiological component were higher in the upper grades (10th–12th grade) than in the lower grades (7th–9th grade) (see Table 5).

**TABLE 5:** Comparison by grade, Mann–Whitney U with Bonferroni correction, of significant differences found in the degree of anxiety.

	**Grade**	**Average range**	**U**	**Z**	p*
Physiological	7th/11th	194.98/239.90	17,782.500	−3.725	<0.001
	7th/12th	170.41/218.83	11,836.000	−4.141	<0.001
	8th/10th	168.08/216.90	13,890.000	−4.296	<0.001
	8th/11th	155.83/216.01	11,623.500	−5.407	<0.001
	8th/12th	134.62/193.16	7,700.500	−5.609	<0.001
	9th/11th	187.68/230.58	16,724.500	−3.636	<0.001
	9th/12th	163.17/208.94	11,161.500	−4.031	<0.001
Total	8th/10th	171.89/211.66	14,595.000	−3.508	<0.001
	8th/11th	164.47/206.53	13,222.000	−3.783	<0.001
	8th/12th	143.66/180.50	9,372.000	−3.528	<0.001

*Bonferroni corrected *p*-value < 0.004.

Analyzing the average score from the previous school year, statistically significant differences were found for all three components and the total TA score (see Table 6).

**TABLE 6:** Difference in test anxiety components and total TA according to academic performance.

**Component**	**H**	**gl**	***P***
Physiological	12.770	4	<0.05
Cognitive	46.898	4	<0.001
Behavioral	118.796	4	<0.001
Total TA	25.712	4	<0.001

In the cognitive component, the mean values of the students who obtained the A mark were lower than those who obtained worse marks. On the other hand, in the behavioral component it is observed that students with marks D, C-, or C+ reached average ranges significantly higher than the rest. Finally, in the total TA, the significantly higher ranks are found in marks D, C-, C+, and B compared to mark A (see Table 7).

**TABLE 7:** Comparison (Mann–Whitney’s U with Bonferroni correction) of test anxiety components between levels of academic performance.

	**Previous grade mark**	**Average range**	**U**	**Z**	p*
Cognitive	**D/A**	184.65/133.39	8,804.500	−4.881	<0.001
	**C**-**/A**	169.35/110.86	5,651.000	−6.056	<0.001
	**C+/A**	249.94/169.96	13,925.000	−6.051	<0.001
	**B/A**	294.87/209.75	19,494.000	−5.515	<0.001
Behavioral	**D/C+**	282.90/226.67	21,978.000	−4.765	<0.001
	**D/B**	365.46/262.43	24,071.000	−8.251	<0.001
	**D/A**	191.51/123.43	7,410.000	−7.563	<0.001
	**C**-**/C+**	257.54/212,43	17,548.000	−3.866	<0.001
	**C**-**/B**	341.38/249.87	18,982.000	−7.316	<0.001
	**C**-**/D**	169.90/111,89	5,795.000	−7.128	<0.001
	**C+/B**	383.32/339.44	55,259.000	−3.617	<0.001
	**C+/A**	240.28/194.28	17,329.000	−4.454	<0.001
Total	**C**-**/A**	160.31/118,15	6,671.000	−4.380	<0.001
	**C+/A**	241.16/181.99	15,608.500	−4.515	<0.001
	**B/A**	291.01/220.90	21,056.000	−4.542	<0.001

*Bonferroni corrected *p*-value < 0.006.

### Test Anxiety, Test Type, and Test Subject

According to the degree of anxiety it provokes in students, the type of exam was ordered as follows: oral classroom test alone with the teacher (*M* = 2.11; SD = 1.25), oral test in front of the class (*M* = 2.63; SD = 1.27), oral presentation in front of a panel (*M* = 2.36; SD = 1.45), oral classwork test (*M* = 2.10; SD = 1.28), essay test (*M* = 1.92; SD = 1.31), full subject test (*M* = 1.21; SD = 1.39), practical test (*M* = 0.97; SD = 1.17), and multiple choice test (*M* = 0.97; SD = 1.14). With respect to the examination subjects the result was math test (*M* = 2.14; SD = 1.46), general knowledge test (*M* = 1.36; SD = 1.20), and physical test (*M* = 1.07; SD = 1.33).

The type or subject of the test is related to the total score and the components of TA with two exceptions in the behavioral component: the individual test and the oral test in front of the teacher (see Table 8).

**TABLE 8:** Pearson correlation coefficient between components of TA and anxiety in different types and subjects of the test.

	**Physiological**	**Cognitive**	**Behavioral**	**Total TA**
Oral test alone with the teacher	0.323**	0.329**	0.016	0.304**
Oral test in front of the class	0.422**	0.438**	0.086*	0.457**
Oral classwork test	0.366**	0.375**	0.130**	0.398**
Multiple choice test	0.321**	0.263**	0.136**	0.313**
Essay test	0.492**	0.533**	0.154**	0.546**
Full subject test	0.431**	0.487**	0.148**	0.489**
Oral presentation in front of a panel	0.305**	0.270**	−0.035	0.299**
Practical test	0.272**	0.200**	0.152**	0.255**
General knowledge test	0.366**	0.358**	0.102**	0.385**
Math test	0.436**	0.445**	0.093**	0.467**
Physical tests	0.163**	0.140**	0.106**	0.164**

**The correlation is significant at the 0.01 level (2 tailed). *Correlation is significant at the 0.05 level (2 tailed).

Next, the influence of sociodemographic and academic variables on each type or subject of examination was analyzed according to the anxiety it generated in the students. In terms of gender, it was observed that girls had significant higher level of anxiety than boys in all types of test.

Considering the grouped age, differences were obtained in oral test in front of the class, oral classwork test, multiple choice test, and essay test. Thereby, only the age group of 15–16 years indicated having more anxiety than 12–14 years old students in a oral test in front of the class (*U* = 52,696; *p* < 0.05). On the other hand, the 15–16 years old (*U* = 76,941, *p* < 0.001) and 17-18 year-old group (*U* = 52,095, *p* < 0.01) scored significantly higher in anxiety than the 12–14 years-old students in the oral classwork test. In the multiple choice test, the 17–18 years old showed more anxiety than the rest of the groups (12-14 years old: *U* = 55,223; *p* < 0.001, and 15–16 years old: *U* = 40,362; *p* < 0.05). Finally, the 12–14 years-old group showed more anxiety than the 17–18 years old in essay test (*U* = 57,172; *p* < 0.05). When establishing correlations between age and anxiety, according to test type, the following correlations appear of significance: oral test in front of the class (*r*_*xy*_ = 0.940, *p* < 0.005), oral classwork test (*r*_*xy*_ = 0.550, *p* < 0.001), multiple choice test (*r*_*xy*_ = 0.115, *p* < 0.001), essay test (*r*_*xy*_ = -0.057, *p* < 0.05), oral test alone with the teacher (*r*_*xy*_ = 0.132, *p* < 0.05) and general knowledge test (*r*_*xy*_ = -0.085, *p* < 0.05).

As a final point, when comparing academic levels, ESO students scored significantly higher in a general knowledge test (*U* = 39,153; *p* < 0.01) and a physical tests (*U* = 12,2285; p < 0.01); while the Bachillerato students indicated having more anxiety when performing an oral classwork test (*U* = 110,815; *p* < 0.01), multiple choice test (*U* = 110,205.500, *p* < 0.001), and oral test in front of the teacher (*U* = 7541; *p* < 0.001).

## Discussion

The main objective of the present study was to examine the differences in sociodemographic variables (gender and age) and academic variables (educational level, grade, mark and type and subject of test) in TA, based on the theory of the triple system of anxiety response proposed by Lang, with an adolescent population.

Firstly, it should be noted that the girls’ higher level of anxiety corroborates the finding verified in the field of anxiety in general and TA in particular (Eman et al., 2012; Putwain and Daly, 2014). In line with our results, the meta-analysis on TA by Von der Embse et al., 2018 concluded that in all grades, from primary to post-secondary education, girls are more anxious. Several explanations for this phenomenon have been proposed (Aydin, 2019; Brandmo et al., 2019): (1) greater sensitivity to social approval leads girls to be more self-demanding, (2) girls have lower expectations of self-efficacy, and (3) girls have a higher perceived threat from the test situation.

In our study we proposed that the higher anxiety levels of girls would be due to the subjective component of TA, i.e., mainly to vegetative overactivation (physiological component) and, to a lesser extent, to excessive worry (cognitive component), while in accordance with Aydin (2019) there would be no difference in the objective component, i.e., in avoidance and escape responses (behavioral component), or if there were, it would be boys that scored the highest. Our results corroborate those of previous studies with adolescent (Rodríguez et al., 2014) and college students (Cassady and Johnson, 2002). The gender gap in primary education widens as children progress through the educational system to post-secondary levels where it tends to decrease (Kurt et al., 2014).

There is a positive relationship between TA and age, such that older adolescents present higher levels of anxiety, similar to what happens in social anxiety disorder where the adolescent is exposed to social evaluation (Olivares et al., 2003). Since age is associated with the grade, and the educational level, students at the top of high school have more TA, especially in the pre-university grades where the higher academic demands may intensify TA.

The relationship of TA with academic performance has been studied mainly in the university population and the data are contradictory. Álvarez et al. (2012) found no relationship among the three components of TA and high school, post-secondary, and college entrance test scores; Ávila-Toscano et al. (2011) found that 100% of low-performing students had significant responses to all three components of TA, while only 7.2, 11.9, and 9.5% of high-performing students had physiological, cognitive, and behavioral TA responses. From the perspective of the theory of the reduction of processing efficiency, Piemontesi and Heredia (2011) found a negative relationship of interference and lack of confidence with TA, but not of worry or emotionality. It is possible that the TA of students with low marks worsens academic performance, but it may also be that the high degree of self-pressurizing of students with high marks interferes with their performance, which would explain the disparity in the results. Academic performance is a complex phenomenon that depends on multiple factors, not only personal factors such as TA, but also variables outside the student’s control such as the teaching method or classroom climate. This issue requires further studies in the adolescent population to shed light on the relationship between TA and academic performance.

The results show differences in the degree of anxiety caused by the type of test. In line with the study by Núñez-Peña et al. (2016), the oral test in front of the class is the most feared situation. This type of test has been linked to social anxiety because the student is exposed to being evaluated not only by the teacher, but also by their peers. In addition to demonstrating academic knowledge, public speaking in the classroom requires the student to possess social and communication skills, which prevent the appearance of fear of negative social evaluation and its possible negative repercussion on performance during the oral test (Laurin-Barantke et al., 2016). The math test is another very feared modality (Carey et al., 2017; Kiliç and Çetin, 2018); it is worth noting, however, that it is associated more with high failure (Wu et al., 2012) than with difficulty in learning (Brown et al., 2020).

Our results are consistent with previous works that revealed that girls are more anxious about taking different types of tests (Rodríguez et al., 2014; Van Mier et al., 2019; Milovanović, 2020), with the exception of the study by Devine et al. (2012), which found no difference according to gender. In the particular case of the math test, the data are contradictory (Sevgi and Arslan, 2020).

This study has several limitations. Firstly, the generalization of the results is limited by the selection of the participants, conditioned by the availability of the educational centers that were willing to allow an investigation to be carried out during the exam period with all the inconveniences this involves. Secondly, the evaluation was limited to questionnaires answered by the students; it would have been desirable, in the framework of the multi-method and multi-source evaluation, to administer other instruments and collect data from other informants such as teachers and parents. Thirdly, it is worth to point out the limitations relative to anxiety assessment before the specific type of tests due to the method employed. Finally, although our study is the first to analyze the relationship of sociodemographic and academic variables with TA, using the adaptation of an instrument for the university population, it would be desirable to develop a specific questionnaire for the adolescent population.

Future studies should extend the research to personal variables, such as the general and anxiety symptoms of the student; family variables, such as the parenting style of the parents; and educational ones, such as the teaching and evaluation of the subjects of study.

Our study highlights the importance of adapting the intervention to the student’s TA profile, according to a prescriptive approach to assessment and treatment of the anxiety disorders in childhood and adolescence (Eisen and Schaefer, 2005; Méndez et al., 2014; Orenes et al., 2017). Additionally, it would be beneficial to prepare risk groups for types of tests that generate high TA, e.g., for girls in the last grades of high school who have to take oral tests in front of their classmates.

## Data Availability Statement

The raw data supporting the conclusions of this article will be made available by the authors, without undue reservation.

## Ethics Statement

The study was reviewed and approved by Ethics Committee of the University of Murcia. Written informed consent to participate in this study was provided by the participants’ legal guardian/next of kin.

## Author Contributions

All authors participated in the design of the study, the analysis and interpretation of the data, the writing of the initial version of the manuscript, and approval of the final version. RT, in addition to these tasks, coordinated the data collection.

## Conflict of Interest

The authors declare that the research was conducted in the absence of any commercial or financial relationships that could be construed as a potential conflict of interest.
